# Individuals with dyslexia use a different visual sampling strategy to read text

**DOI:** 10.1038/s41598-021-84945-9

**Published:** 2021-03-19

**Authors:** Léon Franzen, Zoey Stark, Aaron P. Johnson

**Affiliations:** 1 Department of Psychology, Concordia University, Montréal, Canada; 2 Institute of Neuroscience and Psychology, University of Glasgow, Glasgow, UK; 3 CRIR/Lethbridge-Layton-Mackay Centre de Réadaptation du CIUSSS du Centre-Ouest-de-l’Île-de-Montréal, Montréal, Canada; 4 Réseau de Recherche en Santé de La Vision, Montréal, Canada

**Keywords:** Dyslexia, Reading, Perception, Psychology

## Abstract

Individuals with dyslexia present with reading-related deficits including inaccurate and/or less fluent word recognition and poor decoding abilities. Slow reading speed and worse text comprehension can occur as secondary consequences of these deficits. Reports of visual symptoms such as atypical eye movements during reading gave rise to a search for these deficits’ underlying mechanisms. This study sought to replicate established behavioral deficits in reading and cognitive processing speed while investigating their underlying mechanisms in more detail by developing a comprehensive profile of eye movements specific to reading in adult dyslexia. Using a validated standardized reading assessment, our findings confirm a reading speed deficit among adults with dyslexia. We observed different eye movements in readers with dyslexia across numerous eye movement metrics including the duration of a stop (i.e., fixation), the length of jumps (i.e., saccades), and the number of times a reader’s eyes expressed a jump atypical for reading. We conclude that individuals with dyslexia visually sample written information in a laborious and more effortful manner that is fundamentally different from those without dyslexia. Our findings suggest a mix of aberrant cognitive linguistic and oculomotor processes being present in adults with dyslexia.

## Introduction

Within today’s society, quick and accurate reading skills are essential to participate in societal activities (e.g., education, using social media, communication) and to achieve professional success. Reading is a complex task requiring the eyes, specifically the fovea of the retina, to stop on the written text to successfully encode the sequence of letters that make up each word, and will eventually be assigned meaning (i.e., semantics) during decoding and matching with one’s mental lexicon. The stops in eye movements, termed fixations, are an essential part of reading^1,2^ and a prerequisite for adequate decoding of a text’s content including successful word selection from the mental lexicon^3^. Each fixation is complemented by a brief jump in eye position, termed a saccade, which relocates the fovea to the next part of the text to be processed. Saccades are typically 7–9 characters wide^1^ and take 20–30 ms during reading^4^. The time available for completing a fixation and its associated linguistic tasks, and planning the next eye movement (a saccade) is approximately 225–250 ms per stop^1,4,5^. This duration may be even shorter due to the preview benefit of text appearing outside of the fovea^6,7^. These findings underline the importance of an automatized, rapid interplay of low-level eye movements with higher-level cognitive processing during reading such as lexical, phonological, and semantic processing^1,8,9^.

One group that is well-known to struggle with developing proficient and fast reading skills are individuals with dyslexia. Dyslexia is a language-based, neurobiological specific learning disorder affecting reading, writing, and spelling^10,11^, which persists into adulthood^10,12–14^. Specifically, struggles with accurate and/or fluent word recognition and decoding abilities that typically result from deficits in phonological awareness characterize this disorder^10,11^. Slow reading and a deficit in reading comprehension can be resulting secondary consequences^10,11,15^. An estimated 5 to 20% of the population are affected^11,12,15,16^. Dyslexia’s aetiology remains the subject of a heated debate with proponents attributing the main underlying cause to deficits in a variety of systems associated with reading (i.e., phonological awareness, visuo-spatial attention^17^, magnocellular and cerebellar function^18–21^, or a lack of reading experience^22,23^). While deficits in phonological awareness are considered established^24–28^, other deficits in low-level sensory processing^29–33^ and visual attention remain under scrutiny (e.g.,^34^).

Frequently, researchers who investigate the reading skills and strategies of children and adolescents^35–37^ or adults^38–40^ with dyslexia during sentence reading have focused on reading speed as a measure of performance. These studies report that readers with dyslexia read at a slower rate (i.e., fewer words per minute) compared to readers without dyslexia^35,37–40^. The difference in reading rates between affected and non-affected adults with dyslexia can equate to the difference observed in early readers^39–41^. However, reading speed rates neither provide insight into the cognitive mechanisms nor the visual sampling strategy by which readers with dyslexia may decode written text differently.

Recent evidence stresses that at least one subtype of dyslexia is affected by differences in visual processing^42^, which can be detected within eye movement recordings^43,44^. For example, Nilsson Benfatto and colleagues^43^ were able to reliably distinguish between 9–10 year-old children at high- and low-risk of dyslexia using a classification algorithm operating on one-minute eye movement recordings. Specifically, the duration of fixations, and the number of fixations, saccades and regressions were found to be the most predictive eye movements for differentiating between children at high and low risk of persistent reading difficulties. This finding is in line with previous research showing that readers with dyslexia exhibit longer fixation durations^45–47^, an increased number of fixations^47^, shorter saccades^1,46–48^, and fewer skipped words^3,49,50^. Conversely, the probability of revisiting a previous part of a text (i.e., expressing a regressive saccade also termed regression) has not proven to be reliably different in dyslexia^3,46^. It remains unknown, however, if a similar pattern classification is possible for adults with dyslexia across a range of different texts that are presented in different fonts.

**Figure 1:**
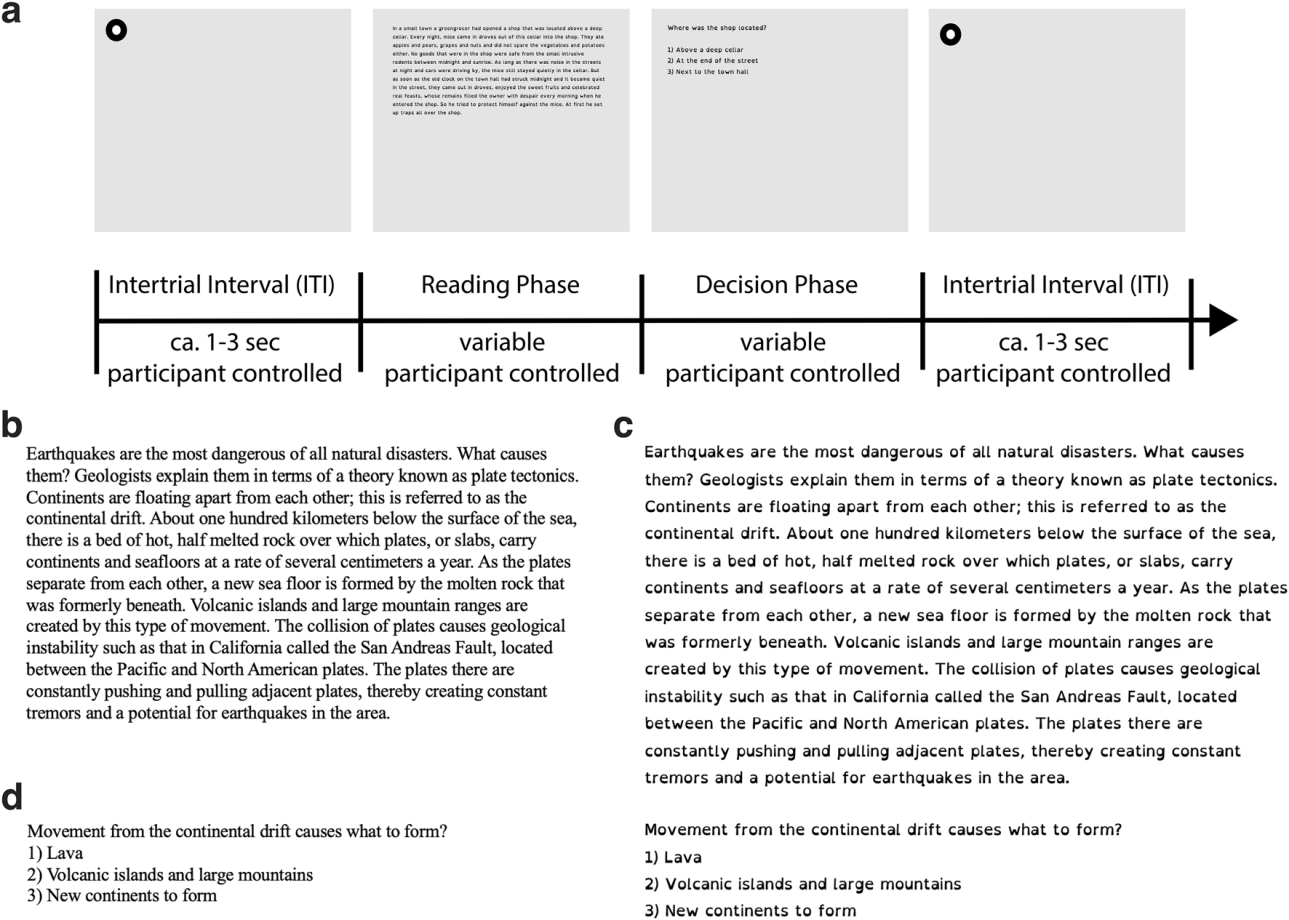
Experimental paradigm and example of stimuli. **(a)** Pictorial depiction of the sequence of events of one trial. The entire paradigm was participant controlled by pressing the space bar. A drift correction circle (the circle was smaller than depicted) directed the participant’s eyes to the starting location of the text. While fixating at it, participants pressed the space bar to get a text to appear that was then read silently once. Immediately after participants finished reading, they were asked to answer a short multiple-choice attention question relating to the content of the preceding text without time pressure. This process was repeated ten times. (**b**) Example of one text and its corresponding attention question (panel d) displayed in Times New Roman font. Note that this text was not presented and just constitutes an example comparable to the texts from the commercial reading assessment (IReST^63^). The original texts are protected. (**c**) Example of the same text and its multiple-choice question displayed in OpenDyslexic font. (**d**) Example of the multiple-choice attention question accompanying the texts shown in panels b and c. Attention questions were always presented in the same font than their preceding text.

Many advances in dyslexia eye movement research have been made in recent decades. Most of our current knowledge about differences in eye movements in dyslexia is provided by researchers investigating either a limited number of eye movement metrics in relation to specific linguistic aspects most often embodied by a target word (e.g.,^51–53^), or is limited by the use of a large variety of often controlled but non-standardized linguistic stimuli in several languages with varying orthographic depth ranging from one character up to one or two sentences^54–57^. Hence, a comprehensive profile of the eye movements of adults with dyslexia during naturalistic reading of standardized texts of multiple sentences remains surprisingly unknown. The development of a comprehensive profile would allow to uncover and quantify potential inefficiencies in visual sampling of text that have not come to light using the aforementioned focused, local approach. Therefore, we aim to devise a comprehensive eye movement account of adult dyslexia by investigating how eye movement patterns of individuals with dyslexia differ from those without dyslexia on global (text-based) and local (word-based) reading measures during an ecologically valid silent paragraph reading task in English (Fig. 1a).

Linguistic parameters such as the difficulty of a text^58,59^, its syntax^60,61^, word length and word frequency^62^ can impact eye movements, highlighting the importance of using standardized and validated stimuli. To this end, we employed the International Reading Speed Texts (IReST^63^). The IReST were developed for standardized reading speed assessment, and consist of ten independent texts, each of about 150 words using novel content. The texts have been equated on a global level for their number of words, syntax, sentence complexity and text difficulty, and were designed to be used in repeated measures within-participant paradigms. Each text was accompanied by one brief multiple-choice question that intended to keep readers attentive and read for comprehension (Fig. 1c,d). The IReST and their accompanying questions were recently validated in a Canadian sample of adult readers attending university^64^. This validation sample was similar in age and education compared to the present study’s sample, though, it was exclusively comprised of adults without reading disability. Perceptual parameters such as the properties of fonts (e.g., spacing) are another aspect that has been shown to affect reading performance^52,65–68^. In an attempt to alleviate the reading struggles of dyslexic readers, designers have developed dyslexia-friendly fonts such as OpenDyslexic^69^ and Dyslexie^70^. These fonts omit serifs, increase inter- and intra-word spacing, and have unique letter strokes. Interestingly, these manipulations have not been found to increase reading speed^36,71,72^. To avoid obtaining an eye movement profile biased by font type, we presented half of the texts in OpenDyslexic (Fig. 1b,c).

Based on previous research (e.g.,^13,73^), we hypothesize that individuals with dyslexia, compared to an age- and education-matched control group without dyslexia, will take longer to read each text and show slower visual processing speed that is in turn linked to one’s reading duration. Reading texts in the dyslexia-friendly font OpenDyslexic is not expected to result in increased reading speed. In terms of eye movements, we hypothesize that readers with dyslexia will express more eye movements (i.e., fixations, saccades, and regressions), longer fixations and shorter saccades. Scanpaths of readers affected by dyslexia are hypothesized to be longer and to differ in their sequence and duration of eye movement events as a result of increases in reading duration.

## Results

In this study, we focused on group-level differences in behavior and eye movements between adults with and without dyslexia. Behavioral analysis included an investigation of the dependent variables reading duration, attention to the text and non-linguistic cognitive processing speed as a function of the two experimental groups (i.e., Dyslexia and Control). Eye movement analyses examined global (i.e., paragraph/trial-based) and local (i.e., word/interest area-based) metrics of eye movement events during reading.

### Behavioral results

We constructed a generalized linear mixed-effects model (GLMM) for analyzing reading duration as a function of the predictors: *group* (Dyslexia and Control) and *font* (Times New Roman and OpenDyslexic), and their *font-by-group* interaction on a single-trial level. The predictor font was also included as a random effect, which was allowed to vary by participant. In addition, the predictors group and font and their interaction were included as random effects, which were allowed to vary by text. This model was based on 601 experimental trials (i.e., one value for median reading duration per trial) collected from all 67 participants. Text number five had to be excluded across all participants due to a stimulus presentation issue (67 trials; 10%). Two further trials from the dyslexia group had to be excluded due to recording issues, which resulted in 601 trials being included in all analyses (for details, see “Methods”).

**Figure 2:**
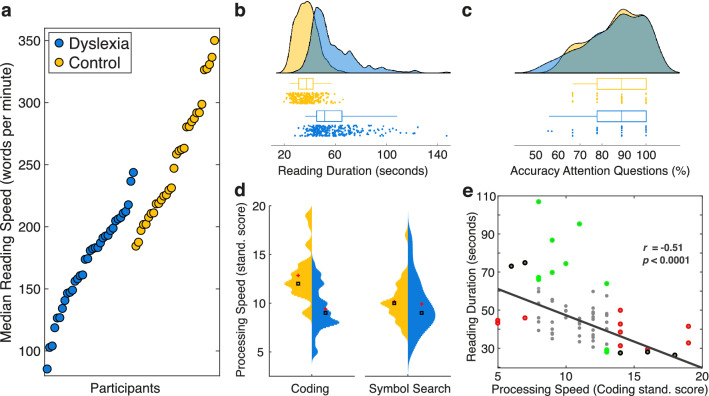
Behavioral results. **(a)** Caterpillar plot of median reading speed (words per minute) per participant sorted in ascending order. Blue dots represent participants of the dyslexia group whereas yellow dots represent participants of the control group. (**b**) Single-trial reading duration in seconds by group. Groups are color-coded as in (**a**). (**c**) Attention to the texts as a measure of reading comprehension. Color notation as in (**a**). (**d**) Cognitive processing speed from the WAIS-IV scale^74^ by group. Higher scores illustrate faster processing speed. The left-hand side (i.e., yellow/light color) of each violin plot depicts scores of the control group; the right-hand side (i.e., blue/dark color) depicts scores of the dyslexia group. Red crosses denote group means, black squares group medians. (**e**) Correlation between reading duration (seconds) and standardized coding processing speed scores across all participants. The shown correlation coefficient (*r*) and p-value resulted from a robust bend correlation analysis (*n* = 67). Colors indicate down-weighted data points: red for data in X, green for data in Y and black for data in X and Y dimensions. In each dimension, 20% of the data points were down-weighted. This figure is best viewed in color.

This GLMM demonstrates significant predictive power of the main effect of group on median reading duration with individuals with dyslexia taking longer to read each text (*X*^2^ = 13.431, *df* = 1, *p* < 0.001; Fig. 2a,b; see Table 1 for detailed model statistics). This speed difference is underlined by a lower words per minute reading rate among readers with dyslexia (*Median*_Dyslexia_ = 178.09, *Median*_Control_ = 248.18; two-sided independent sample *t*-test: *t*(65) = 20.51, *p* < 0.0001; *g* = 1.67, 95% CI_g_ = [1.486, 1.858]; Fig. 2a). However, this model yields neither a significant improvement in reading duration with font (*X*^2^ = 1.41, *df* = 1, *p* = 0.235) nor a significant font-by-group interaction (*X*^2^ = 0.446, *df* = 1, *p* = 0.504).

**Table 1:** Generalized linear mixed-effects model fixed effect parameter estimates.

Parameter	Estimate	CI 2.5%	CI 97.5%	SE	t	p
Font	1.097	− 0.6593	2.8531	0.896	1.224	0.221
**Group**	**− 21.696**	**− 32.3832**	**− 11.0091**	**5.453**	**− 3.979**	** < 0.0001**
Font * Group	− 1.207	− 4.7307	2.3167	1.798	− 0.671	0.502

Model predicting reading duration. Effects with significant predictive power after post-hoc likelihood-ratio (*X*^2^) model comparisons in bold.

Secondly, the multiple-choice questions presented immediately after reading each text served as an incentive for participants to read each text for comprehension—hence, constituting an indicator of attention. Both groups demonstrate attention to the texts clearly above chance level (control: *t*(31) = 17.67, *p* < 0.0001; BF_10_ = 5.1457 × 10^14^; Min = 66.67%; dyslexia: *t*(34) = 15.96, *p* < 0.0001; BF_10_ = 2.2968 × 10^14^; Min = 55.56%; all two-sided paired *t*-tests; Fig. 2c). That is, two participants in the dyslexia group scoring at 55.6% and 57.1%, nine participants across both groups showing performance at 66.7%, and the remaining 56 (out of 67) participants answered more than 75% of the attention questions correctly, with 31.3% of all participants answering all questions correctly. Crucially, our data show no significant evidence for a difference between both groups (two-sided independent samples *t*-test: *t*(65) = − 0.34, *p* = 0.7349; BF_10_ = 0.2635; Fig. 2c). Altogether, this analysis demonstrates that both groups paid attention to the reading material, as their performance is better than chance across all trials.

Thirdly, we examined if there were any non-linguistic cognitive processing speed differences between our two groups using two subcomponents from the Wechsler Adult Intelligence Scale (Coding and Symbol Search^74^). This analysis was motivated by previous reports of links between reading speed and slower cognitive processing speed in individuals with dyslexia^75^. We find that individuals with dyslexia exhibit slower processing speed on the Coding test (two-sided independent samples *t*-test: *t*(65) = 5.88, *p* < 0.0001; *g* = 1.422, 95% CI_g_ = [0.895, 1.973]; Table 2; Fig. 2d), but not on the Symbol Search test (independent samples *t*-test: *t*(65) = 0.399, *p* = 0.69; *g* = − 0.1, 95% CI_g_ = [− 0.382, 0.577]; Table 2; Fig. 2d). Our data further show a negative correlation between coding speed and reading duration across all participants (*r*_65_ = − 0.51, *p* < 0.0001, 95% CI_r_ = [− 0.680, − 0.315]; Fig. 2e) suggesting that, in the present study, participants with better coding ability (i.e., faster number related cognitive processing speed) exhibit shorter reading duration. Symbol search speed did not correlate with reading speed across participants (*r*_65_ = 0.12, *p* = 0.33, 95% CI_r_ = [− 0.106, 0.35]). Although these single measures both probe visual processing speed, their separate interpretation warrants caution, since they may not provide a full representation of one’s processing capabilities as outlined in their use^76^.

**Table 2:** Descriptive statistics of group characteristics.

Measure	Mean (SD)		Median (Variance)	
	Control	Dyslexia	Control	Dyslexia
Age	22.38 (2.7)	23.54 (6.22)	22 (7.27)	21 (38.67)
Diagnosis age	NA	12.69 (6.43)	NA	10 (41.28)
Adult Dyslexia Checklist	31.31 (3.33)	52.14 (10.55)	31 (11.06)	51 (111.36)
Reading speed (wpm)	254.76 (47.93)	171.32 (38.37)	252.85 (2296.8)	180.91 (1472.1)
Symbol search	10.16 (2.02)	9.91 (2.83)	10 (4.07)	9 (8.02)
Coding	12.84 (2.53)	9.40 (2.27)	12 (6.39)	9 (5.13)

Control group (*n* = 32), dyslexia group (*n* = 35). Visual cognitive processing speed measured in standardized scores based on age as assessed by the Symbol Search and Coding subtests of the WAIS-IV^74^. A score of 10 equates to the population average on these tests.

These results appear to be the consequence of the control group showing coding speed above the general population average while the dyslexia group shows performance slightly below the general population average (*M*_population_ = 10, *M*_Control_ = 12.84, *M*_Dyslexia_ = 9.40; Table 2; Fig. 2d). One reason for these results might be that the coding task encompasses working memory performance to some degree^77^. Memorizing digit-symbol pairs only shown at the top of the page more quickly may constitute a strategy for achieving a higher score on this test. Hence, although not explicitly testing working memory performance, these results may be indicative of working memory deficits in adults with dyslexia when compared to a similarly educated non-dyslexia group—in line with previous reports^78–82^—and their role in achieving age- and education-appropriate reading speed. However, we find no general visual processing speed deficit in dyslexia as both the Coding and Symbol Search test need to be considered in unison^76^.

In short, our behavioral results show a sustained level of attention to the stimulus material throughout the majority of this study by most participants. Though, readers with dyslexia exhibit generally slower reading speed in line with previous reports. One potential explanation of the observed reading speed deficit might be a difference in the skills probed by the non-linguistic Coding processing speed test but not a general visual processing speed difference.

### Eye movement profile

**Figure 3:**
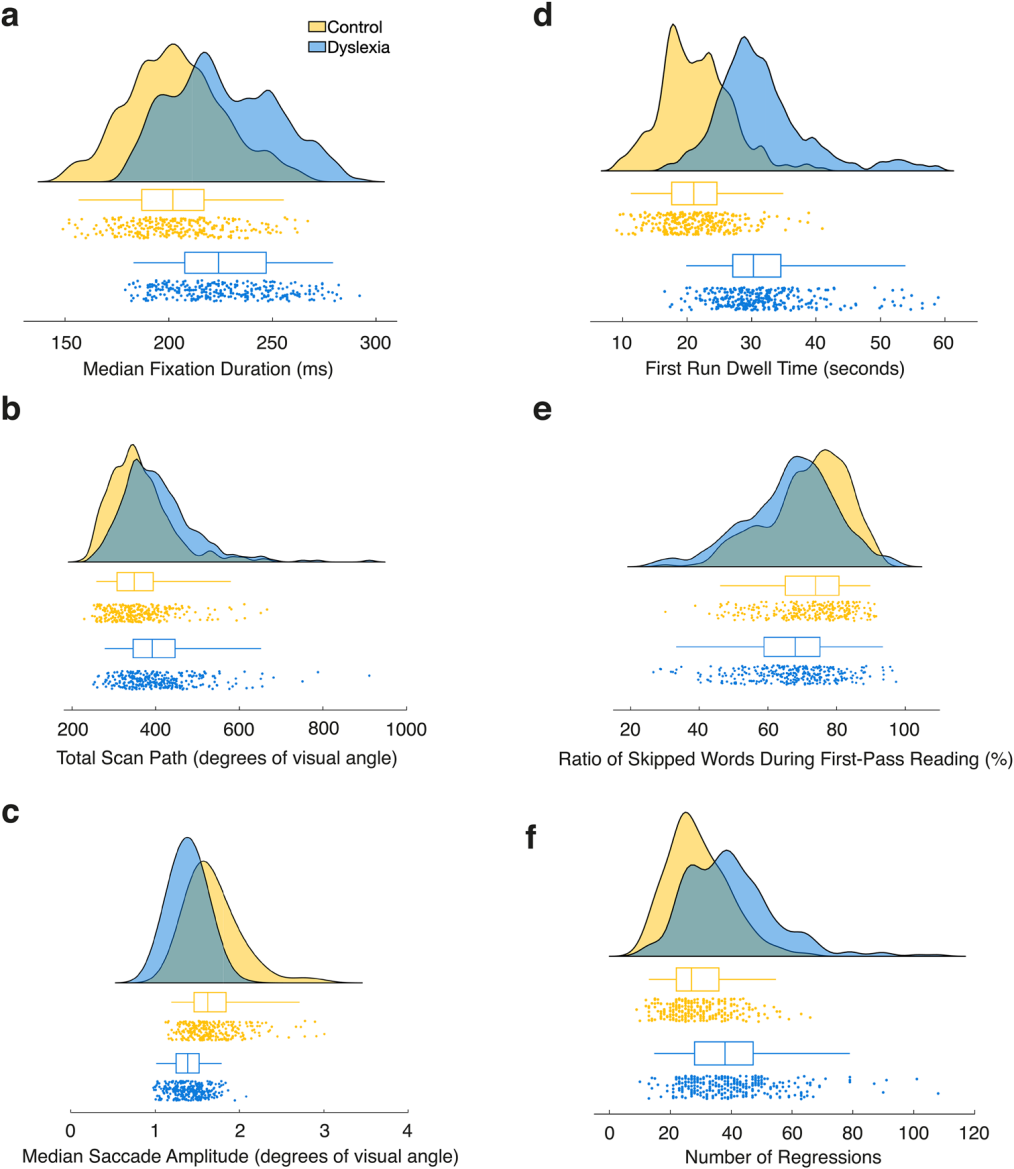
Group comparison of selected traditional eye movement metrics. Plots display trial-based eye movement metrics that showed significant differences between groups. Each panel depicts the group comparison collapsed across fonts as a raincloud plot for the respective metric. Kernel density plots depict the frequency of occurrence of a value while scatterplots display the underlying values as in one average value per trial. Boxplots indicate the median, upper and lower quartile, and whiskers the 95% CI. Blue (dark) color represents data of the dyslexia group whereas yellow (bright) color depicts data of the control group. (**a**) Median fixation duration in milliseconds. (**b**) Total scan path in degrees of visual angle. (**c**) Median saccade amplitude in degrees of visual angle. (**d**) First run dwell time in seconds. That is, the sum of all fixation durations during a first visit to a word if it has not been visited or skipped before. (**e**) Ratio of words that were skipped during first-pass reading. This excludes all fixations on a word that occurred after a regression to a previous word was completed. (**f**) Number of regressions. That is, leftward saccades to preceding words that have already been visited or skipped.

**Figure 4:**
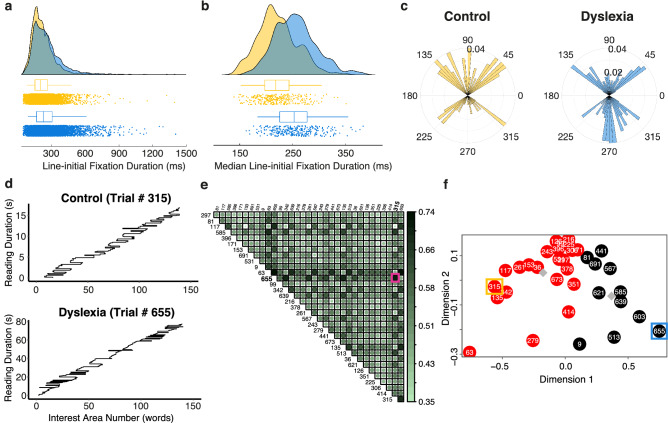
Contemporary eye movements typical and atypical for reading. **(a)** Duration of all identified line-initial fixations by group. That is, the very first “valid” fixation on the first two words of a line not followed by a leftwards corrective saccade. Boxplots indicate the median, upper and lower quartile, and whiskers the 95% CI. Blue (dark) color represents data of the dyslexia group whereas yellow (bright) color depicts data of the control group. (**b**) Median line-initial fixation duration per trial by group. Color scheme and box plot properties as in (**a**). (**c**) Group comparison of the frequency of directional deviations by angle across all trials depicted as polar histograms. Saccade angle and frequency are depicted in bins of 2.75° width. Only saccade angles between ± 35° and ± 145° (displayed as 35/145° and 215/325°) qualified as directional deviations. 0° equals horizontal rightward direction of reading. All other saccades were excluded from this analysis. **d)** Example of two scanpaths after reading the same text (IReST #10) displayed in Times New Roman font. Scanpaths are depicted over time (y-axis) and word-based interest areas (x-axis). Top scanpath depicts trial by a control participant, whereas the bottom scanpath depicts a trial expressed by a reader with dyslexia. (**e**) Pairwise, trial-based similarity matrix illustrating differences in spatial and temporal fixation patterns upon normalization by reading duration. Higher scores (darker color) denote higher dissimilarity. Pink square indicates the Scasim dissimilarity score for the two trials shown in (**d**). (**f**) Map of clusters of trials in the spatial domain (using the Euclidean distance metric) after multidimensional scaling. Red and black colored circles denote trials allocated to separate clusters. Grey diamonds indicate the centers of these clusters. Trials framed in yellow and blue represent the spatial equivalent of the same trials whose scanpaths are depicted in panel d. Colors mark groups.

**Figure 5:**
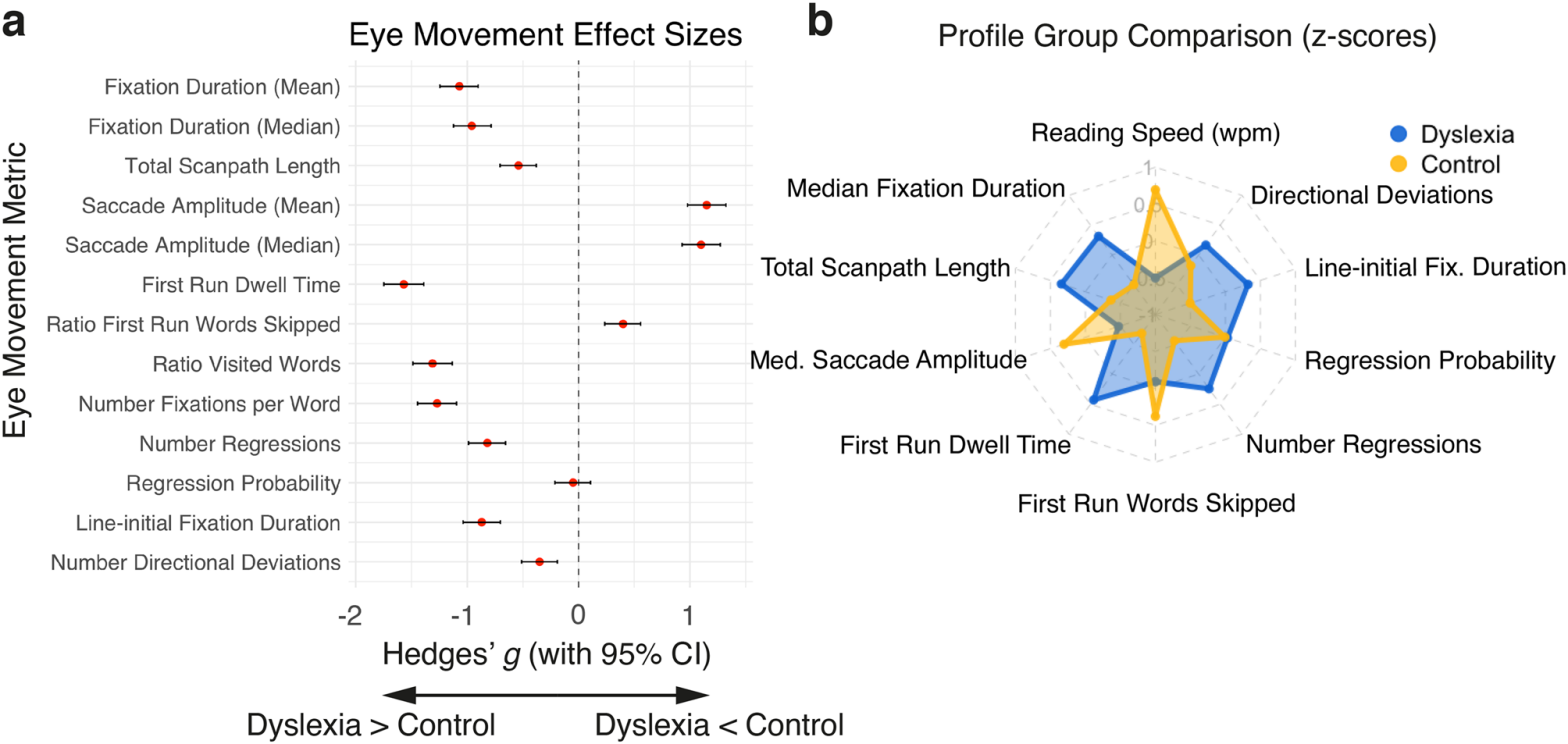
Summary of effects and visual sampling strategy group profiles. (**a**) Effect sizes and their 95% confidence intervals (CIs) of the effect of group on eye movement metrics. Positive effect sizes (i.e., Hedges’ *g*) illustrate a higher number of events, longer duration or distance or a larger ratio among control participants. A negative effect size illustrates the opposite effect. Red dots denote the effect size and black bars the 95% CI for each effect size. CIs computed using the exact analytical method as implemented in the measures of effect size toolbox^83^. Effect sizes were considered significant if the 95% CI did not include zero. (**b**) Radar plot depicting overall group differences in the eye movement and reading profile given selected metrics. Plots depict group averages after all trials of each measure were normalized (i.e., z-scored) for comparability. Counter-clockwise direction follows presentation order as in (**a**). If two variants of the same metric were present in panel a, only one of them is displayed on the radar plot for simplicity.

This study aims to devise a comprehensive characterization of the eye movement profile of individuals with dyslexia during natural paragraph reading. This profile comprises eye movement metrics traditionally examined in the field (Fig. 3), and other more recent metrics such as line-initial fixation duration, scanpath similarity, and specific saccades atypical for reading (Fig. 4). The reading-related metrics covered in this study include global (i.e., trial-/text-based) and local (single-word based) metrics. To establish the significance of a group difference in the frequentist sense, we use unbiased effect sizes^83^ (i.e., the 95% confidence interval of Hedges’ *g* not including zero; denoted as *g* in the text; Fig. 5a). A negative effect size indicates a longer duration or larger number exhibited by the dyslexia group and vice versa. High collinearity between some metrics included in our analyses did not allow for the use of a meaningful linear regression approach. In general, where applicable, we report group means alongside group medians to complement this robust measure of central tendency and ensure comparability to previous literature.

Traditionally, average fixation duration and saccade length have been investigated on a global trial-level as indices of cognitive processing effort. In an effort to replicate previous findings, we computed the same metrics showing that readers with dyslexia stop longer as illustrated by longer mean and median fixation duration (*g*_Mean_ = − 1.07, 95% CI_g_ = [− 1.244, − 0.902]; *g*_Median_ = − 0.96, 95% CI_g_ = [− 1.124, − 0.786]; 83% of readers with dyslexia show this effect; Figs. 3a and 5a,b), and scan more area of the text as indicated by a longer total scanpath (*g* = − 0.54, 95% CI_g_ = [− 0.706, − 0.380]; Figs. 3b and 5a,b). While scanning the text, the dyslexia group exhibits shorter average saccade amplitude (i.e., eye movement “jumps” between fixations; *g*_Mean_ = 1.15, 95% CI_g_ = [0.978, 1.323]; *g*_Median_ = 1.10, 95% CI_g_ = [0.930, 1.273]; 100% of readers with dyslexia show this effect; Figs. 3c and 5a,b), and increased variance (i.e., standard deviation) in these saccade amplitudes (*g* = 0.70, 95% CI_g_ = [0.495, 0.823]). This pattern of results suggests that adults with dyslexia employ a more laborious visual sampling strategy on a global text-level. Longer fixation durations and shorter saccade amplitudes indicate that this group spends more time on information uptake and analysis per stop (i.e., fixation), while also potentially taking up less information per unit of time. A similar pattern of eye movements has previously been associated with individuals who are learning to read^2^, and those who are considered poor readers^56^. The observed longer total scanpaths among readers with dyslexia are a logical consequence of this group’s substantial increase in reading duration.

Global metrics, however, do not provide more detailed information on specific elements of the visual sampling strategy in relation to single words such as how many of these words get actively fixated and how often. To address these questions, we complemented the reported global metrics with metrics based on the definition of interest areas around single words. We find that individuals with dyslexia spent more time (*g* = − 1.57, 95% CI_g_ = [− 1.75, − 1.39]; Fig. 3d and *First Run Dwell Time* in Fig. 5a,b), and skip fewer words (*g* = 0.40, 95% CI_g_ = [0.235, 0.557]; Fig. 3e and *Ratio First Run Words Skipped* in Fig. 5a,b) during first pass reading (i.e., the sum of all first fixations on a word in reading direction excluding any revisits or skipped words). In line, readers with dyslexia fixate on more words in a given trial when all fixations are examined (*g* = − 1.312, 95% CI_g_ = [− 1.488, − 1.135]; *Ratio Visited Words* in Fig. 5a), and stop more frequently per word on average (*g* = − 1.27, 95% CI_g_ = [− 1.445, − 1.095]; *Number Fixations per Word* in Fig. 5a). Further, revisits of earlier parts of a text (i.e., leftward saccades to a preceding word formally called regressions) are a substantial and frequent part of natural reading. We observe that readers with dyslexia express more regressions per text (*g* = − 0.82, 95% CI_g_ = [− 0.989, − 0.656]; Figs. 3f and 5a,b). However, given the increase in the number of saccades as a result of longer reading durations, this increase in the number of regressions yields no significant difference in the probability of making a regression across an entire text (*g* = − 0.05, 95% CI_g_ = [− 0.213, 0.107]; Fig. 5a,b).

Furthermore, since the control group shows coding processing speed above the population average, we examined this group’s link between fast coding speed and the traditional eye movement metrics reported above. This analysis shows no correlation between coding speed and any of the reported eye movement metrics (absolute range *r*_30_ = 0.016–0.32, all *p*s > 0.05), which suggests that faster coding speed does not systematically affect the eye movements of readers without dyslexia. We also observe no correlation for the dyslexia group (absolute range *r*_33_ = 0.019–0.14, all *p*s > 0.05).

Taken together, our results on traditional eye movement metrics corroborate previous findings from investigations with readers affected by dyslexia. They demonstrate that these readers examine a given text more slowly and in smaller steps, even without accounting for any revisits of previous words (Fig. 5b). Since efficient reading was found to be characterized by skipping over many words (up to 90%^3^) during the first rightward scanning of a text in reading direction (termed, first-pass reading), the observed pattern strongly suggests that inefficiencies are introduced by processing less content simultaneously as well as slower information uptake and longer cognitive processing times of text. Crucially, these findings are based on data obtained from natural reading of standardized texts consisting of multiple lines.

### Further contemporary metrics of ocular movements during reading

Recently, additional metrics have been proposed to differentiate between oculomotor deficiencies and cognitive, linguistic factors underlying longer fixation times^84^. Line-initial fixations are one such metric. They constitute the first fixation on one of the first words of a line that is not followed by a leftwards correction within the same line. Uniquely, line-initial fixations do not allow the reader early access to a word’s coarse visual orthographic percept due to absent parafoveal preview. Hence, they have been proposed as an unconfounded indicator of linguistic processing time^84,85^. By contrasting groups on the duration of line-initial fixations, we find these to be longer in the dyslexia group on a single-fixation (*g* = − 0.33, 95% CI_g_ = [− 0.383, − 0.276]; *Median*_Dyslexia_ = 231 ms, *Median*_Control_ = 202 ms; Fig. 4a), and single-trial level (*g* = − 0.81, 95% CI_g_ = [− 0.972, − 0.64]; *Median*_Dyslexia_ = 256 ms, *Median*_Control_ = 216 ms; 89% of readers with dyslexia show this effect; Figs. 4b and 5b). Parker and colleagues^84^ reported an effect in the same direction when comparing accurate line-initial fixation durations between children and adults without dyslexia, with children showing longer fixation durations. This finding adds to the evidence indicating that readers with dyslexia take longer to process the visual and linguistic information sampled during a fixation. It further supports the notion that the visual sampling strategy of readers with dyslexia resembles the strategy of early readers without dyslexia.

To ensure that we selected line-initial fixations accurately, we compared their duration to the overall median fixation duration of a trial. Previous research shows that fixation duration decreases as readers move their eyes towards the end of a line^84,86^. As expected, the identified line-initial fixations are of longer duration than all fixations considered together (*g*_Dyslexia_ = 0.87, 95% CI_g_ = [0.673, 1.071]; line-initial_Dyslexia_ = 256 ms; All_Dyslexia_ = 224 ms; *g*_Control_ = 0.57, 95% CI_g_ = [0.361, 0.768]; line-initial_Control_ = 216 ms, All_Control_ = 202 ms). We find this increase in line-initial fixation duration to be larger in the dyslexia group (*g*_Groups_ = − 0.37, 95% CI_g_ = [− 0.526, − 0.204]; 32 vs 14 ms). The same pattern of eye movement results reported above holds true when analyzing only trials whose attention questions were answered correctly.

### Specific divergence from a regular visual sampling strategy

Besides the presented group differences on global and local eye movement metrics, we noticed a clear divergence from a regular left-to-right visual sampling strategy among readers with dyslexia. To quantify these divergences of eye movements that we consider atypical for reading, we examined saccades with angles that would not be expected during the natural reading flow (henceforth, directional deviations).

During left-to-right reading of text most saccades will either be expressed in a horizontal rightward direction in line with the expected reading direction or in mostly horizontal leftward direction as revisits of preceding text or in an occasional return-sweep saccade to the beginning of the next line. Yet, not all observed saccades would satisfy any of these typical categories of eye movements, since their expressed angle deviates substantially from the ones expected as part of the usual reading flow. We examined the number of saccades with an angle that considerably deviates from the expected pattern of saccade angles during typical reading (i.e., angles between 35° and 145° upwards and − 35° and − 145° downwards from the horizontal reading plane). To avoid that falsely programmed return-sweeps were mistaken for directional deviations, we excluded a number of saccades that could be attributed to other eye movements typically involved in reading such as blinks, accurate and inaccurate return-sweeps (for details, see “Methods”). These corrections resulted in 522 saccades being identified as directional deviations.

Readers with dyslexia express directional deviations more than twice as often per trial on average (*g* = − 0.48, 95% CI_g_ = [0.316, 0.641]; *Mean*_Dyslexia_ = 1.003, *Mean*_Control_ = 0.441, *var*_Dyslexia_ = 2.08, *var*_Control_ = 0.61; 69% of readers with dyslexia show more directional deviations per trial than the control group’s average; Figs. 4c and 5b), which signals a more frequent loss of place at unexpected points during the reading process. Remarkably, in the dyslexia group most of the identified directional deviations were directed straight downwards, whereas this pattern was virtually reversed in readers without dyslexia (Fig. 4c). Since even a brief scanning of the area of text just below the current fixation seems rather unintuitive from a cognitive perspective, this finding raises the question whether these directional deviations are the result of occasional issues with oculomotor control previously reported in dyslexia^29,31–33,87–93^.

The aforementioned differences in eye movements are part of the overall visual sampling strategy of text during reading, termed a scanpath. To investigate whether readers with and without dyslexia differ only on some eye movement metrics or rather use a divergent overall visual sampling strategy, we complemented the previous analyses with a computational similarity analysis of the overall scanpath of each trial. To this end, we quantified the temporal and spatial similarity of the fixations of all scanpaths employing a version of the *Scasim* analysis^94^. The aim of this trial-based analysis was to identify clusters of trials with similar scanpath patterns, while achieving independence of the observed group differences in reading time. To identify whether trials of readers with dyslexia were more (dis)similar to those of other readers with dyslexia, we compared the number of trials associated with each group within a given cluster. Similarity scores and clusters were computed separately for each text of the IReST battery and font type, as this coordinate-based analysis is highly sensitive to differences in spacing such as those introduced by text displayed in differently spaced font types (Fig. 1b,c). In this study, trials were equally split between Times New Roman and OpenDyslexic font types. Additionally, all trials were normalized by their reading duration to avoid the introduction of trivial differences between scanpaths of different lengths.

Pairwise scanpath similarity scores showed that trials of participants with dyslexia differed from those of participants without, even though participants read identical texts (Fig. 4d–f). Upon normalizing, trial-by-trial similarity scores indicated that dyslexic participants spend a substantial amount of the time (between ~ 34 and 83%) looking at different places on the identical paragraph and/or for different durations compared to their non-dyslexic counterparts (Fig. 4e). These similarity scores were subsequently transferred to the spatial domain (for details, see “Methods”), where we find that the optimal number of group-independent clusters ranges between two and five clusters per text-font pair. Trials of each group were predominantly allocated to separate clusters, and demonstrate a significant difference of association for about 75% of text-font pairs (*p* < 0.05; for detailed statistics, see Table S3 in the supplementary material). Thus, we find that readers with dyslexia sample identical texts using a different sequence of fixations (i.e., fixating different locations on the text and/or for different durations) than non-dyslexics—even when differences in reading time are accounted for.

To summarize, our findings demonstrate that readers with dyslexia use a generally more laborious and inefficient visual sampling strategy during natural reading. The virtually opposite pattern of directional deviations between groups points towards the existence of occasional deficiencies in oculomotor control that result in dyslexic readers losing their place more often. Replicating previous findings, their laborious strategy is characterized by longer average and line-initial fixation duration, prolonged first run dwell time as well as shorter saccade amplitude and fewer skipped words. Contrarily, the probability of revisiting preceding words was comparable between groups. This pattern of eye movements suggests that prolonged time for cognitive, linguistic processes such as word decoding, lexical access, and/or phonological decoding underlies the behavioral difficulties associated with dyslexia such as substantially slower reading speed; but not an increased need for resolving semantic or syntactic ambiguities through reanalysis of prior text. Altogether, these results indicate that an interplay of linguistic and oculomotor factors underlies the reading struggles in adults with dyslexia.

## Discussion

In this study, we used eye-tracking to devise a comprehensive eye movement profile of the visual sampling strategy of adult readers with dyslexia during naturalistic reading of standardized multi-sentence texts in English (IReST^63^). Here, combining traditional and contemporary eye movement metrics, we show fundamental differences between readers with and without dyslexia on all but one of the examined metrics. These results, in combination with substantial decreases in reading speed, illustrate a laborious and more effortful reading strategy in adulthood, resembling a pattern observed in beginning^2^ and poorer readers^56^.

The idea that eye movements differ between readers with and without dyslexia is not new. Rayner^1,48^ was among the first to report different eye movements during reading based on anecdotal case studies with only three dyslexics. His investigations were followed by numerous cross-sectional studies using separate samples of readers with dyslexia, and largely varying stimuli in languages with different orthographic depth (for reviews, see^2,95^). This variety of stimuli, typically consisting of hand-picked single words or short sentences that impose artificial task demands on the reader rather than allowing for an ecologically valid natural reading scenario, constitutes an issue in the field^96^. The use of standardized and validated multi-sentence texts remains scarce in the literature.

In this work, we were particularly interested in reconciling many previously separate accounts of differential eye movements using the same sample of individuals with dyslexia while also examining specific indicators of oculomotor deficiencies during natural reading. Our results replicate previous findings of differential eye movements in children and adults with dyslexia such as longer fixation durations, fewer skipped words during first-pass reading and repeated fixations on the same word^1,3,43–50,97^. Oculomotor control has commonly been investigated using a variety of non-linguistic saccade tracking and fixation stability tasks^29,33,87,88,90,91,93^. Here, we show that specific saccades atypical for reading can be detected during natural reading. A twofold likelihood of expressing such a saccade, termed directional deviation, indicates signs of occasional oculomotor deficiencies in dyslexia—in line with previous reports^29,31–33,87–93^.

Given that the eye movement profile of children with dyslexia during paragraph reading has previously been exploited for dyslexia screening^43,44^, it is worth asking whether the inclusion of metrics on separate levels of granularity (i.e., the local single-word and global paragraph level) in adults improve our understanding of eye movements in dyslexia? Our approach differs crucially from these two screening studies on several points. Firstly, both studies were conducted with children around the age of 10 using recordings obtained from reading only one non-validated text with short lines. Secondly, these studies aimed to identify the most parsimonious model that classified recordings accurately as stemming from a child with or without dyslexia. This focus on reducing complexity in the data precluded devising a comprehensive profile, and may have resulted in overlooking smaller but informative differences such as directional deviations. Thirdly, this model-focused approach did not allow for addressing specific hypothesis-driven questions using targeted measures such as line-initial fixations. Hence, these child studies and our adult study complement each other by establishing an eye movement profile of dyslexia at different ages that consists of a diverse set of metrics.

To answer the central question about the source of the reading struggles of individuals with dyslexia, all differences across the entire profile need to be considered. These differences can be explained in the context of established eye movement models that are able to simulate a wide range of reading related patterns including fixation duration, multiple fixations on a word, parafoveal preview benefit, regressions, and spill-over effects—E-Z Reader^62,98–100^, SWIFT^101^, OB1-Reader^6^. While these models were devised to explain the reading process of typical readers, they can also provide insight into the processes of beginning^100,102^ and dyslexic readers^3,99^. Particularly, the extensive research on the E-Z Reader model, and its prior application to data obtained from readers with dyslexia, makes it an interesting model for our study. It is a serial processing model which posits that processing of the fixated word occurs in four separate stages, beginning as soon as the preceding word (N) is fixated on and attention is allocated. First, information about the upcoming word (N + 1) is extracted from the parafovea during pre-attentive visual processing. Second, a *familiarity check* is performed upon fixation. The length of this familiarity check is dependent on the word’s frequency and length. Once a word is processed for lexical familiarity, it signals the initiation of an eye movement. Third, *completion of full lexical access* leads to a shift in attention to the next word (N + 1) in the form of a saccade. Fourth, an integration of the identified word in the syntactic context is carried out simultaneously and may trigger a regressive saccade, if a syntactic or semantic conflict is detected^62,98,99^.

In the case of individuals with dyslexia who present with longer fixation durations, as seen in the current study, this model posits that these individuals experience slower lexical access, associated with increased lexical processing demands. Where skilled readers require less time to perform the usually fast familiarity check (i.e., finding a match for the letter string making up a word), individuals with dyslexia do not seem to be able to carry out this process equally fast. The dyslexia group also expressed shorter saccades and skipped fewer words during first-pass reading—in line with previous findings^1,3,48^. These findings, in combination with a higher frequency of fixating on the same word repeatedly, corroborate the notion of needing to process each word or even its sub-components individually, and for longer, when reading for comprehension. Prolonged line-initial fixations of individuals with dyslexia in our study provide more evidence for delays in lexical access (stages two and three). These first fixations on a line do not benefit from any parafoveal preview benefit (stage one) resulting in the sole reliance of information sampled during this fixation for word identification purposes.

While a deficit in lexical processing can explain longer fixation durations for individuals with dyslexia, a deficit in parafoveal processing could likewise explain increased fixation durations, shorter saccade amplitudes and fewer skipped words. The preview benefit takes advantage of orthographic information from parafoveal vision such as word length and word familiarity. It is linked with a reader’s perceptual span, which is defined as the number of distinct characters from which useful information can be acquired in parallel across the fovea and parafovea^52,103,104^. Should individuals with dyslexia present with such a deficit, removing or having a reduction in this preview benefit could also result in the need for longer processing (i.e., fixation durations) when the next word is being fixated on simply due to reduced pre-processing of its orthographic percept^105^. Such a smaller perceptual span has previously been associated with reading speed^106,107^, and reported in dyslexia^2,108^. This deficit can occur independently of a phonological deficit^109^, however, previous evidence is inconclusive^110,111^. In the present study, line-initial fixation duration can serve as an indicator of parafoveal processing when compared to all other fixation durations across a sentence. The former are usually longer due to absent parafoveal preview while the duration of all other fixations decreases towards the end of a line^84–86^. These line-initial fixations were longer for all readers compared to the median fixation duration across a trial. This difference was larger for readers with dyslexia, which suggests that these readers need even more processing time when no preview benefit is available. In other words, readers with dyslexia do not appear to be disadvantaged when the preview benefit for words to the right of a fixation is available. This result demonstrates that a reduction in the perceptual span is unlikely the explanation of the general increases in fixation duration, as seen in this study’s sample with dyslexia.

Another conceivable reason for explaining dyslexics’ reading struggles is that readers with dyslexia encounter increased semantic and syntactic processing difficulties. These difficulties would lead to revisits of previous words, as illustrated by the frequently observed increase in the number of regressive saccades^1,45,48,58,89^. In the E-Z Reader model, regressions are conceptualized as difficulty with integrating the identified word within the greater sentence context—occurring during a fourth post-lexical integration stage. In line with previous literature, we observed a greater number of regressions per trial in individuals with dyslexia. Vagge and colleagues^89^, similar to our work, reported this result for a study that also found slower reading speed. However, the higher number of regressions in both studies may simply be a result of longer reading durations, since we did not find a difference in the probability of making a regressive saccade in relation to the overall number of saccades of a trial. This absent regression probability difference replicated an earlier report with dyslexic adults^3^. With the E-Z Reader model in mind, our findings speak against a deficit in the post-lexical integration stage of reading leading to the reading struggles observed in the present study^99^.

Although the traditional eye movement metrics reported in this work suggest that prolonged lexical processing time underlies the slower reading speed and visual profile in dyslexia, the role of oculomotor deficiencies is far from being established in the literature^19,34,95,112^. Their role has been at the center of the on-going dyslexia debate. To investigate potential oculomotor deficiencies during natural reading, as opposed to saccadic tracking and fixation stability tasks, we examined saccades that are unexpected or atypical in the reading flow of a skilled reader. We should consider horizontal rightward, leftward and diagonal return-sweep saccades reading related. One may even include saccades pointing upwards in this list, since they might constitute a sudden double-checking of previously read content. However, readers with dyslexia jumped away from some fixations in directions atypical for reading such as straight downwards more often than their counterparts. We found the directional pattern of these atypical eye movements to be virtually reversed between groups. This indicates that common eye movements such as blinks were not falsely identified as directional deviations in either group, as they should have resulted in a similar pattern of directional deviations (for details, see “Methods”). The nature and frequency (i.e., ~ 1 per trial) in the dyslexia group suggest that these directional deviations may be a residual of oculomotor deficiencies that persist, but only occur occasionally, in adults with dyslexia attending higher education. Thus, differences in oculomotor control appear unlikely to be the origin of their reading struggles—at least in the present sample of individuals with dyslexia.

One conceivable concern of the present study’s design is that the observed eye movement profile could be simply a result of texts that were too difficult for readers with dyslexia, since increases in text difficulty were reported to lead to more eye movements in dyslexic children^58^, and a pattern similar to the one observed in this study in readers with^1^ and without dyslexia^2^. However, given that the IReST are designed at a grade six reading level and all of the participants in the present sample had previously attended or were attending higher education at the time of participation, unsuitable text difficulty is very unlikely to be the reason for the observed differences.

A second concern is that we did not observe a ceiling effect in the responses to our multiple-choice questions—with two participants performing just above chance and 31.3% of all participants answering all questions correctly. Although there are multiple reasons as to why not all participants answered all questions correctly, one is the very specific nature of some questions, which makes those somewhat more challenging for some readers. Importantly, we found no evidence for a difference between the two groups. Such a difference could have raised concerns about it exerting effects on the eye movement contrasts in this study.

Third, we aimed to provide a comprehensive descriptive profile that can be paired with predictions from established models of eye movement control. However, this profile itself may not be used to confirm causality of the deficits underlying the general struggles of individuals with dyslexia. Particularly, given that the present study tested adults and administered only a hypothesis-driven assessment of selected cognitive skills such as visual processing speed, more information could prove useful in future studies to pinpoint the exact origins of the reading struggles and their causal links. It is further conceivable that differences on reading tasks in adults originate from less or reduced reading experience as opposed to neurobiological consequences of dyslexia per se^23^. Therefore, the presented eye movement profile may serve as a basis for more targeted investigations in the future probing the specific nature of the presumed deficit in lexical access and its direct causal link to the cognitive mechanisms of the reading difficulties. In this respect, the extensive debate on the aetiology of dyslexia is in dire need of more longitudinal investigations starting in kindergarteners to increase the likelihood of being able to establish such causal links reliably through observing the pre-reading state and its change over time.

In summary, the presented eye movement profile of adults with dyslexia demonstrates a laborious and effortful visual sampling strategy when reading multiline paragraphs of text. Specifically, the combination of prolonged fixation duration, shorter saccade amplitude and fewer skipped words suggests deficits in the linguistic processing components of reading such as fast and efficient access to the mental lexicon. Longer line-initial fixation durations were particularly indicative of prolonged lexical analysis. On the contrary, we did not find convincing evidence for a perceptual span deficit or increased difficulties in the semantic or syntactic post-lexical processing stage of reading. An increased number of eye movements atypical for reading shows that the eyes of readers with dyslexia occasionally move to seemingly random places on a page. Hence, occasional oculomotor deficiencies should not be categorically dismissed in dyslexia.

## Methods

### Participants

We tested 73 participants: 35 adults with an official diagnosis of dyslexia, and 38 without symptoms of dyslexia. Six individuals from the control group were excluded from all analyses due to large inaccuracies during the calibration procedure (best eye with average error > 0.5° and max error > 1.3°). Hence, the final data analysis was conducted on 67 participants: 35 with dyslexia (female = 23, *Mean*_age_ = 23.54, *SD*_age_ = 6.22) and 32 without (female = 32, *Mean*_age_ = 22.38, *SD*_age_ = 2.7).

To delineate between control participants who experience dyslexia symptoms but have not been given an official diagnosis, and to get a measure of severity of dyslexia symptoms at the time of participation, all participants completed the Adult Dyslexia Checklist^113^. This checklist assesses aspects of literacy, language, word finding, and organization skills on a scale of 1–4 (i.e., *rarely / occasionally / often / most of the time*). As specified by the original authors, a score of 45 or more points indicates mild to severe dyslexia symptoms^113^. We used a score of ≤ 40 points as a conservative cut-off for our control group, with all formally diagnosed dyslexics allocated to the dyslexia group.

Participants were matched on age and level of education. They were either current or former college or university students at anglophone institutions in Canada. Since participants were recruited in Montréal, a bilingual English-French city, our sample comprises both bilingual and monolingual English speakers (bilingual_Dyslexia_ = 18, monolingual_Dyslexia_ = 17; bilingual_Control_ = 9, monolingual_Control_ = 23). To avoid introducing a language effect, we compared bilingual to monolingual participants and found no evidence for a difference in reading duration in the dyslexia group (*t*(33) = 1.829, *p* = 0.0765; *g* = 0.6, 95% CI_Dyslexia_ = [− 0.064, 1.264]; BF_10_ = 1.16) nor the control group (*t*(30) = − 0.5, *p* = 0.6212; *g* = − 0.19, 95% CI_Control_ = [− 0.94, 0.56]; BF_10_ = 0.4). We neither observed a language effect regarding attention to the text in the dyslexia group (*t*(33) = 1.375, *p* = 0.1784; *g* = 0.45, 95% CI_Dyslexia_ = [− 0.206, 1.108]; BF_10_ = 0.67), and insufficient evidence in the control group (*t*(30) = − 2.241, *p* = 0.0326; *g* = − 0.86, 95% CI_Control_ = [− 1.634, − 0.071]; BF_10_ = 2.16). Based on these findings, groups were collapsed across language for all analyses. Written informed consent was obtained from all participants. Participants could choose between receiving $10 or course credit as compensation. This study adhered to the Canadian Tri-council Policy on ethical conduct for research involving humans^114^, and obtained approval by the Concordia University Human Ethics Research Committee (certificate: 30003975).

### Stimuli

In this study, we used the International Reading Speed Texts as stimuli^63^. These are 10 texts that have been equated based on their number of words, syntax, sentence complexity and text difficulty (*M*_text_ = 153.6 words, range_text_ = 140–160; *M*_sentence_ = 8.9, range_sentence_ = 8–11), and are designed for use as repeated measures within participants. Additional to their original validation in a UK sample, the IReST texts have been validated in an adult Canadian sample comparable in age and education to the present sample (validation sample: *n* = 25; *M* = 21.72, *SD* = 4.94, range = 1–41)^64^. None of the participants in this validation study reported having reading or attention disabilities. No participant took part in both studies conducted at Concordia University (i.e., the Canadian validation and present eye-tracking study). As is common practice in reading studies, each text was accompanied by one short multiple-choice question with three options of which one was correct (for examples, see Fig. 1b–d). This attention question was presented immediately after reading a text. These questions have been used in other reading studies^64,115^. Five texts and their accompanying multiple-choice questions were displayed in Times New Roman font, with the other five texts and questions being displayed in the specific dyslexia font OpenDyslexic^69^. Sentence start and end words were identical between the font versions resulting in identical launch and landing interest area sites across font conditions. To equate for differences in physical text size, we varied the nominal font sizes using 20-point size for Times New Roman and 18-point size for OpenDyslexic trials. Text height was comparable. Every text and its respective multiple-choice question were presented in the same font. Due to copyright restrictions by virtue of the text being a commercial reading assessment, we are unable to provide the reader with the 10 IReST paragraphs.

The Wechsler Adult Scale of Intelligence’s Symbol Search and Coding subtests were administered to assess processing speed abilities of all participants (WAIS-IV^74^). Importantly, both subtests use non-linguistic stimuli. In the Symbol Search task, participants are shown two target symbols and are instructed to identify both of the target symbols within the adjacent search group. This task involves no working memory as the symbols change for each trial (i.e., by horizontal search group of five symbols). Contrarily, the Coding task may involve aspects of working memory^77^. Participants are shown numbers 1–9 and their unique corresponding symbol at the top of the page. Here, the task is to draw the corresponding symbol associated with each number below a sequence of numbers. The WAIS has an internal consistency score of 0.87–0.98 on processing speed index tasks. The interscorer agreement ranges from 0.98 to 0.99, and intraclass correlation from 0.91 to 0.97^116^. Correlations between scores on tests that measure similar constructs were in the 0.8 range on criterion-related validity measures^117^.

### Procedure

The Adult Dyslexia Checklist^113^ was first completed, after which the experimenter administered the WAIS Symbol Search and Coding tasks^74^. Subsequently, participants were calibrated to the eye tracker (EyeLink 1000) by looking at a series of 9 equally spaced dots on grey background across the screen (using the SR EyeLink’s inbuilt 9-point calibration procedure with targets in the default locations). A validation procedure using the same 9 points in a randomized order confirmed the accuracy of the eye tracker’s calibration measurements, with participants’ better eye needed an accuracy of < 0.5 degrees on average and no point exceeding 1.3 degrees of maximum error. Upon successful calibration, participants were instructed on screen and verbally to read the 10 texts once thoroughly in silence. They may go back and reread words or entire sentences but once they had reached the end of the text and attempted to start from the beginning they were immediately stopped by the experimenter. Each text was presented separately (five in TNR font, five in OpenDyslexic). We displayed texts in the upper half of the screen with 83.57 characters per line on average and left alignment (Fig. 1a). Once a participant finished reading, they pressed the spacebar, subsequently a multiple-choice question concerning the previously read text appeared. We randomized the presentation order of these 10 texts within-subjects (using the *randperm* function in MATLAB). An additional text with similar linguistic properties, comparable to the text in Fig. 1b,c and a multiple-choice question was presented as a practice trial in Arial regular font. After completing the reading portion, participants filled out a questionnaire concerning their experience reading the 10 texts as well as demographic information such as age, mother tongue and education (see supplementary information for full questionnaire). In total, the experiment lasted between 30 and 45 min.

### Apparatus

Stimuli were presented and data collected using an iMac (2011 27″ i7, 16 GB RAM) with an external monitor (View Sonic G225fb 21″ CRT, 1024 X 768 pixel resolution, 100 Hz refresh rate). A chin rest was used to stabilize head position at a distance of 70 cm from the screen. Eye position was acquired non-invasively using a video-based eye movement monitor (EyeLink 1000 running host software version 4.56, SR Research, Ottawa, Ontario).

### Eye tracking analysis

Eye movement data were recorded at a sampling rate of 1000 Hz and stored for offline analysis. DataViewer’s inbuilt algorithms (version 4.1.1, SR Research, 2019, Ottawa, Ontario) were used for the pre-processing of fixations, saccades, and blinks, forming reading-related interest areas and trial-based aggregate measures. An interest period that excluded the first and last 300 ms of each trial was defined in DataViewer to avoid contaminating this analysis with reading unrelated events at the very beginning and end of each trial. The duration of fixations that spanned any of these two cut-off time points was trimmed. Further, the minimum saccade amplitude was set to 0.5°, the fixation merging amplitude to 1°, and the minimum fixation duration to 50 ms. Fixations separated by a blink were not merged. Instead we removed fixations immediately before and after a blink. Fixations beyond display bounds (i.e., the entire screen) were excluded. In general, we analyzed only data of one eye per participant and excluded all samples that were identified by any of the aforementioned criteria from all further analyses. These analysis parameters help to remove outliers caused by random eye movements that are unrelated to reading.

The interest area analysis was word based in that one interest area was associated with each word including five pixels of padding around all sides of a word. A background RGB threshold of less than 350 was chosen to fill gaps between interest areas. Although all fixations were drift corrected by the drift value obtained at the start of each trial, we manually adjusted all fixations of a trial vertically (13.9% of all analyzed trials) if visual inspection showed that fixations exhibited an obvious vertical offset across all lines of a text resulting in them lying on interest area boundaries. Importantly, we neither moved single fixations separately nor adjusted fixations horizontally.

Results of the offline analysis with DataViewer (version 4.1.1, SR Research, 2019, Ottawa, Ontario) were exported for use with custom scripts in MATLAB (version 2020a, The MathWorks Inc., 2020, Natick, Massachusetts). There, we calculated all measures split by experimental conditions (i.e., group and font). We excluded all trials presenting text number five in either font due to a stimulus presentation issue and to avoid any bias (10% of all trials). A further two trials had to be excluded due to recording issues. To quantify and compare the effect of group (i.e., Dyslexia vs Control) in detail, we computed unbiased signed between-group effect sizes (*g*) and their respective 95% confidence intervals separately for each eye movement metric (using the *mes* function of the Measures of Effect Size Toolbox^83^ and its *exact analytical* method for determining confidence intervals). Following frequentist logic, a significant effect of group was presumed when the 95% confidence interval of an effect size did not include zero. In our design, positive effect sizes represent a higher number or ratio of the respective eye-tracking metric for the control group compared to the dyslexia group and vice versa (Fig. 5a). As well, we estimated the probability density function corresponding to selected eye movement metrics whose between-group comparison yielded a significant effect size employing kernel density estimation in MATLAB (using the *raincloud_plot* function^118^). In doing so, we created a probability density heat map for all selected eye movement metrics.

In addition to traditional reading eye movement metrics, we also examined line-initial fixations. These fixations have been proposed to be able to dissociate binocular coordination from linguistic analysis of text/words^85^. We identified the first fixation on any of the first two words of a line that was not followed by an undersweep corrective saccade to the left of this fixation as accurate line-initial fixation. Group differences within these fixations were then quantified using Hedges’ g.

Furthermore, we compared the two experimental groups on saccades that are unlikely or unexpected during typical reading behavior/flow. Such saccades were the ones showing radical angular shifts with an angle between ± 35° and ± 145°, which we termed directional deviations. These saccades could not be classified as (in)accurate forward, regression, undersweep, return-sweep or blink saccades. In the computation of these directional deviations, we excluded angles of rightward and leftward saccades typically and frequently involved in reading. All saccades with a blink before, during or after the directional deviation saccade in question, as identified by DataViewer’s built-in algorithm (version 4.1.1, SR Research, 2019, Ottawa, Ontario), did not qualify either. Further, saccades representing correctly programmed return-sweeps to the beginning of the next line were excluded^85^. Return-sweeps were identified by finding potential directional deviations that were launched within 3 words of the final word of a line and landed within the first three words (i.e., interest areas) of the next line. Last but not least, incorrectly programmed return-sweeps (so-called “undersweeps”^84^) were excluded from this analysis as well, since they are not assumed to be involved in on-going linguistic processing^86^. These had to be launched from any of the final four words of a line.

As an overall measure of scanpath similarity, we computed the *Scasim* metric^94^. This trial-based metric compares the location (x-, y-coordinates) and duration of all fixations that make up a scanpath and can be computed using the *scasim* function provided by the first author’s GitHub repository^119^. The resulting score represents a value of dissimilarity. We normalized all scores by their respective reading duration to avoid confounds due to large differences in reading duration. Scasim scores are computed on a pairwise trial-by-trial basis. Since this measure uses the x-,y-coordinates of a fixation’s location, we computed Scasim scores per IReST and font a text was displayed in. This was necessary as differences in font led to words of the same text being displayed in slightly different locations. Trials were analyzed across groups to begin with. However, to quantify scanpath similarity between readers with and without dyslexia, spatial maps of scanpaths were fit on similarity scores using Euclidean distances (*dist* function) and non-metric multidimensional scaling (*isoMDS* function from the *MASS* package^120^). Subsequently, we used the optimal number of clusters, a result of a calculation of Gaussian mixture models paired with the Bayesian Information Criterion^121^ (*mclustBIC* function of the *mclust* package^122^), as the number of clusters in a k-means clustering procedure (*kmeans* function). Since the determined clusters were still comprised of group-independent trials, we employed chi-square and Fisher exact tests of association analyzing whether trials in each cluster belonged to the same or a different experimental group. The entire Scasim analysis was conducted in RStudio^123^.

### Statistical analysis

To investigate potential differences in reading duration as a function of dyslexia (i.e., group factor) and font, we used a generalized linear mixed-effects model (GLMM). Eye movement data was primarily analyzed using unbiased effect sizes (i.e., Hedges’ g; denoted as *g* in the text) and their exact analytical 95% confidence interval^83^. For selected eye movement metrics, we also computed the proportion of participants in the dyslexia group whose trial-based average showed performance in line with their group when compared to the average of all participants in the control group (i.e., above or below the control group’s mean).

The GLMM analysis was performed by means of the *lme4* package^124^ and the *bobyca* optimizer in RStudio^123^. *Reading duration* was specified as a continuous dependent variable, and examined, using a *gamma* model in the family argument of the *glmer* function, as a function of the two categorical predictors: *group* (i.e., Dyslexia and Control), and *font* (i.e., Times New Roman and OpenDyslexic), and their interaction on a single-trial level. The GLMM included the maximal random effects structure justified by the experimental design^125^. They included all main effects and interactions of our two predictors, group and font, as well as by-subject and by-item random intercepts and random slopes for all relevant main effects. We excluded random correlations for this model. The 95% confidence intervals were calculated for all $\upbeta$ estimates (using the *broom* package and *Wald* method in RStudio^123^). We accounted for small imbalances in trial numbers of the predictors’ levels by entering all predictors in mean-centred form (deviation coding). All entered predictors were checked for collinearity (using the *cor* function and model output). Lastly, we used post-hoc likelihood-ratio (*X*^2^) model comparisons to quantify the predictive power and exact significance level of all initially significant or trending effects (i.e., *p* < 0.1) revealed by the GLMM.

To examine potential effects of non-linguistic visual cognitive processing speed on reading duration (i.e., the WAIS subscale scores), particularly in light of a difference in reading duration between experimental groups, we correlated the standardized scores for both processing speed measures with reading duration across participants. This and all other correlations were computed employing robust bend correlations and the default of 20% bending in each direction^126^.

To investigate each groups’ attention to the reading material separately, we first compared their results on our multiple-choice attention questions against chance (i.e., 50%) using two separate two-sided one-sample *t*-tests as well as a Bayes Factor analysis. We also contrasted the two experimental groups against each other by means of a two-sided independent samples *t*-test, under the assumption that both groups would pay equal attention to the reading material. In determining significance, all *t*-tests were Bonferroni-corrected by the number of *t*-tests evaluating this dependent variable (*n* = 3). The Bayes Factor analysis allowed us to quantify the strength of the evidence in support of the null hypothesis of no difference when compared against 0 or between groups. Due to a lack of previous research using Bayes Factors in this area, we used an uninformed prior for the Bayesian analyses with a Cauchy width of 0.7.

## Supplementary information

Supplementary information.

## Data Availability

Data supporting this work is available from the project’s Open Science Framework repository [https://osf.io/3r8gx/].

## Code availability

Code to reproduce selected figures and analyses is available from the project’s Open Science Framework repository [https://osf.io/3r8gx/].
