# Supplementary material for: Individuals with dyslexia use a different visual sampling strategy to read text
Source: Sci Rep. 2021 Mar 19;11:6449. doi: 10.1038/s41598-021-84945-9 (PMC7979812; doi:10.1038/s41598-021-84945-9)
Supplement: Supplementary file 1 — Supplementary information. [file 41598_2021_84945_MOESM1_ESM.pdf]

Supplementary Materials

Individuals with dyslexia use a different visual sampling strategy to read text

Léon Franzen [1,2*], Zoey Stark[1], & Aaron P. Johnson[1,3,4]

[1] Department of Psychology, Concordia University, Montréal, Canada
[2] Institute of Neuroscience and Psychology, University of Glasgow, Glasgow, United Kingdom
[3] CRIR/ Lethbridge-Layton-Mackay Centre de Réadaptation du CIUSSS du Centre-Ouest-de-l'Île-de-Montréal
[4] Réseau de Recherche en Santé de la Vision.

[*] leon.franzen@mail.com

Text #	Font	Design	Test	X^2	*df*	Cramér's V	Alt. Hypothesis	*p*
**1**	**TNR**	**4 x 2**	**Fisher**				**2-sided**	**< .0001**
**2**	**TNR**	**2 x 2**	**chi-square**	**12.725**	**1**	**0.631**		**< .001**
**3**	**TNR**	**2 x 2**	**chi-square**	**10.72**	**1**	**0.598**		**0.001**
4	TNR	3 x 2	Fisher				2-sided	0.0548
**6**	**TNR**	**2 x 2**	**chi-square**	**6.0942**	**1**	**0.452**		**0.008**
**7**	**TNR**	**2 x 2**	**Fisher**				**odds ratio ≠ 0**	**0.0184**
**8**	**TNR**	**2 x 2**	**Fisher**				**odds ratio ≠ 0**	**0.0184**
**9**	**TNR**	**5 x 2**	**Fisher**				**2-sided**	**0.0137**
**10**	**TNR**	**2 x 2**	**chi-square**	**8.601**	**1**	**0.503**		**0.003**
**1**	**OD**	**2 x 2**	**chi-square**	**11.765**	**1**	**0.588**		**< .001**
2	OD	2 x 2	chi-square	1.821	1	0.231		0.177
**3**	**OD**	**3 x 2**	**Fisher**				**2-sided**	**< .0001**
4	OD	2 x 2	Fisher				odds ratio ≠ 0	0.1026
**6**	**OD**	**3 x 2**	**Fisher**				**2-sided**	**0.0016**
**7**	**OD**	**2 x 2**	**chi-square**	**4.136**	**1**	**0.354**		**0.042**
**8**	**OD**	**3 x 2**	**chi-square**	**17.376**	**2**	**0.726**		**< .001**
9	OD	2 x 2	chi-square	0	1	0		1
10	OD	2 x 2	chi-square	2.346	1	0.271		0.126

Table 3. Results of tests of association. Tests performed on cluster counts resulting from the scanpath similarity analysis (Scasim). Results obtained using a chi-square test of association between the number of trials in a cluster of trials with similar scanpaths belonging to either the dyslexia or control group. Where not applicable due to zero counts of a group for a given cluster, the Fisher's exact test was computed as an alternative but only returns a p value. The "Alt. Hypothesis" column refers to the alternative hypothesis used by Fisher's exact test. The Yates' continuity correction was applied to the Cramér's V effects size when a chi-square test was computed. All statistical tests were computed separately for each text-font pair of the IReST. Tests with significant results are highlighted in bold.

Questionnaire administered after the completion of the eye-tracking experiment

Welcome page

UNIVERSITÉ Concordia UNIVERSITY

Thank you for participating in this eye-tracking study. We would like to ask you a couple of questions about the experiment and yourself.

Questions only to be answered by the experimenter

I. Subject ID: #
II. Tracked eye
    a. Left
    b. Right
    c. Both
III. Dominant eye
    a. Left
    b. Right
    c. None

Questions to be answered by the participants

1) How comfortable did reading OpenDyslexic feel to your eyes?
   This is OpenDyslexic
   a. 7-point Likert scale

2) How comfortable did reading Times New Roman feel to your eyes?
   This is Times New Roman
   a. 7-point Likert scale

3) Which of the two font types you encountered during the experiment do you prefer?
   a. OpenDyslexic
   b. Times New Roman

4) Overall, did you find the paragraphs presented difficult to understand?
   a. 7-point Likert scale

5) Overall, did you find the comprehension questions difficult to answer?
   a. 7-point Likert scale

6) Did you feel stressed at any point throughout the experiment?
   a. Yes
   b. Yes, a little at times
   c. No, not at all

7) Please rate your stress level during the experiment.
   a. 7-point Likert scale

8) Do you suffer from visual stress occasionally?
Visual stress refers to a condition characterised by the inability to see comfortably and without distortion when looking at a still standing stimulus, e.g. text. Symptoms are visual fatigue, perceived excessive light sensitivity, headaches from exposure to disturbing visual patterns and several kinds of perceptual distortion such as blurring, fading, or flickering of the visual stimulus. (Uccula et al., 2014)

   a. Yes, almost daily
   b. Sometimes
   c. No, not at all
   d. Only when reading

9) Do you like to read fiction?
   a. 7-point Likert scale

10) Do you like to read academic journal articles?
   a. 7-point Likert scale

11) Would you describe yourself as a regular reader?
By regular we mean reading text heavy formats such as academic journal articles, fiction or non-fiction books multiple times a week.

   a. Yes
   b. No

12) Overall, did you find the paragraphs presented easy to understand?
   a. 7-point Likert scale

13) Overall, did you find the comprehension questions easy to answer?
   a. 7-point Likert scale

Demographics

14) How old are you?

15) What is the current or former field/subject of your studies?

16) What year of your studies are you in?
   a. 1st
   b. 2nd
   c. 3rd
   d. 4th
   e. 1st Master
   f. 2nd Master
   g. PhD
   h. Alumni

17) Have you been given an official diagnosis of dyslexia at some point in your life?
   a. Yes
   b. No

18) If you have been given an official diagnosis of dyslexia, at what age was this diagnosis given?

19) Do you encounter any of the following problems today?

Choose as many as you like

- A Slow reading
- B Slow writing
- C Reading causes tiredness
- D Making frequent spelling mistakes
- E Blurring of lines
- F Blurring of letters
- G Skipping of letters during writing
- H Skipping of words during reading
- I Losing place or missing out on lines when reading
- J Re-reading of entire lines or paragraphs to understand them
- K Going back to a word multiple times
- L Being very forgetful in daily life without regular reminders
- M Difficulty concentrating for longer than an hour
- N Getting easily distracted
- O Difficulties with numbers
- P Difficulties with maths and calculations
- Q Difficulties with rote memory (learning by repetition)
- R Difficulties in learning a foreign language
- S Difficulties in organising your thoughts on paper
- T Difficulty in finding the right word to say
- U None of the above

20) What is your native language?

a. English
b. French
c. Bilingual

21) Which language do you use more often?

a. English
b. French
c. I use both interchangeably to the same extent

22) How many languages do you speak fluently including your native language?

a. 1
b. 2
c. 3
d. 4 or more

23) How many foreign languages have you been formally taught in school or university?

a. 1
b. 2
c. 3
d. 4 or more

24) What country are you a citizen of?

a. Canada
b. United States of America
c. Other

25) If you selected other, please specify your country of citizenship. Otherwise ignore this question.

26) If you selected other, please specify how long you have been living in Canada for. Otherwise ignore this question.

Please fill in a number in months (1 year = 12 months)
